# New data on freshwater psammic Gastrotricha from Brazil

**DOI:** 10.3897/zookeys.60.495

**Published:** 2010-10-07

**Authors:** André R. S. Garraffoni, Thiago Q. Araujo, Anete P. Lourenço, Maria Balsamo

**Affiliations:** 1 Universidade Federal dos Vales do Jequitinhonha e Mucuri, Departamento de Ciências Biológicas, Campus II, Rodovia BR-367, CEP: 39.100-000, Diamantina, MG, Brazil; 2 Dipartimento di Scienze dell’Uomo, dell’Ambiente e della Natura, Università di Urbino, Campus Scientifico, loc. Crocicchia, Urbino, Italy

**Keywords:** freshwater Gastrotricha, Macrodasyida, Chaetonotida, biodiversity, meiofauna

## Abstract

Current knowledge of freshwater gastrotrich fauna from Brazil is underestimated as only two studies are available. The present communication is a taxonomic account of the first-ever survey of freshwater Gastrotricha in Minas Gerais State. Samplings were carried out yielding six species of three Chaetonotidae genera: Aspidiophorus cf. pleustonicus, Ichthydium cf. chaetiferum, Chaetonotus acanthocephalus, Chaetonotus heideri, Chaetonotus cf. succinctus, Chaetonotus sp., and also an undescribed species belonging to the genus Redudasys (incertae sedis): this is the first finding of specimens of Redudasys outside of original type locality. These preliminary observations suggest that the knowledge of the biodiversity of Gastrotricha in the Minas Gerais State, as well as in the whole Brazil, will certainly increase as further investigations are undertaken, and that freshwater Macrodasyida may be more common than previously thought.

## Introduction

Gastrotricha are aquatic free-living microinvertebrates (< 1 mm), with a worldwide distribution in freshwater, estuarine, and marine benthic habitats where they are an important component of the benthos and periphyton (Hochberg and Litvaitis 2000; Balsamo and Todaro 2002; Balsamo et al. 2005, 2008). Although many species are common and occasionally abundant, freshwater gastrotrichs are still insufficiently known, possibly due to their microscopical size, body fragility, which make their study very difficult (Hochberg and Litvaitis 2000; Balsamo and Todaro 2002; Balsamo et al. 2005, 2008). However, despite the minute body size, they are recognized to have both a complex anatomy and life cycle (Weiss 2001).

The taxon consists of nearly 750 named species grouped into two orders, Macrodasyida and Chaetonotida (but see Kieneke et al. 2008), which are greatly different in morphology, reproductive biology and ecology (Balsamo and Todaro 2002; Balsamo et al. 2008; Todaro and Hummon 2008). Macrodasyidacomprise about 300 worm-like species, all interstitial in marine and estuarine habitats except for the two freshwater ones recorded only from their type locality (Hummon and Todaro, 2010): Marinellina flagellata Ruttner-Kolisko, 1955 (Austrian river Ybbs)and Redudasys fornerisae Kisielewski, 1987 (Brazilian dam on the savannah near São Carlos city). The roughly 450 species of Chaetonotida are smaller, tenpin-shaped, and colonize marine, brackish and especially freshwater habitats, where two thirds of the species can be found.

The biodiversity of the Gastrotricha fauna in Brazil is still underestimated (Kisielewski 1991; Marques and Lama 2006; Garraffoni and Araujo in press) because, until now, only few studies have focused on the diversity and distribution of this taxon both in fresh waters (Kisielewski 1987, 1991) and in marine waters (Todaro and Rocha 2004, 2005).

Regarding the freshwater habitat, Kisielewski (1991) reported 14 genera (including three new genera), and 59 species (26 of which new species), from various regions of São Paulo State (cities of São Paulo and São Carlos; Juréia Ecological Reserve), of Mato Grosso do Sul State (city of Corumbá), and Pará State (cities of Belém and Benevides), and from different habitats, such as ponds, reservoirs, rivers, puddles in the tropical forest, mangrove and estuaries. In this study, the author stressed that the diversity of Brazilian fauna of inland-water Gatrotricha appears unusually high, and recommended further faunistic, detailed studies. However, no survey was done later on (Garraffoni and Araujo in press).

Thus, the aim of the present study is to provide the first records of the Gastrotricha fauna from the State of Minas Gerais. This is the first of a series of surveys that will be realized as an effort to increase the taxonomic and biogeographic knowledge of the Brazilian Gastrotricha, with special emphasis on the State of Minas Gerais. Furthermore, with the aim to stimulate new research on this group in Brazil, Garraffoni and Araujo (in press) prepared a taxonomic key for all Brazilian freshwater and marine Gastrotricha, and listed the main morphological characters used to identify species as a glossary with terminologies in Portuguese.

## Material and methods

Samples of the upper sediment were taken from 7 distinct stations located along two small watercourses and one river near Diamantina city at an altitude of 1300 m: Soberbo (-18.193919; -43.570286), Água Limpa (-18.214431; -43.617211), and Preto River (-18.130619; -43.337647). Other sampling locations were: an unnamed stream in the Itambé Peak (-18.397222; -43.328889), at an altitude of 1680 m, and unnamed stream in Cabral Mountains (-17.767694; -44.286000), at an altitude of 1209 m, and an unnamed stream, near Gouveia City, (-18.530139; -43.898833; -18.538667; -43.898000), at an altitude of 1174 m. Gastrotrichs were extracted after repeated washing of small amounts of sediment with 2% MgCl2 aqueous solution. Living individuals were located by examining the supernatant under an Olympus SZ40 stereomicroscope at 40x magnification, and were removed by micropipette to a glass slide. Further observations and photographies were done under a Zeiss Photomicroscope equipped with differential interference contrast optics (DIC) and an Olympus CH30 microscope without DIC.

The morphological study and the identification of gastrotrichs were performed using the terminologies and identification keys presented in Kisielewski (1987), Balsamo and Todaro (2002), Balsamo et al. (2005) and Todaro and Hummon (2008). The descriptions followed the convention of Hummon et al. (1992), whereas the locations of some morphological characters along the length of the body were given in percentage units (U) measured from anterior to posterior. In-group systematization of Chaetonotus and Ichthydium followed Balsamo et al. (2009).

Descriptions of putative new taxa are beyond the scope of the present study, and their definitive affiliation will be made at the end of the ongoing taxonomical surveys in forthcoming papers. However, we provide a photograph of each taxon and the measurement of the main structures, for the benefit of researchers working in the same area who might find them in the meantime.

All adult formalin–glycerin whole-mounts specimens are kept in the meiofauna collection of the senior author at the Universidade Federal dos Vales do Jequitinhonha e Mucuri.

## Taxonomy

### Order Chaetonotida Remane, 1925

**Family Chaetonotidae Gosse, 1864**

#### Genus Aspidiophorus Voigt, 1904

##### Aspidiophorus pleustonicus

Kisielewski, 1991 cf.

Fig. 1 Table 1

Aspidiophorus pleustonicus  – Kisielewski (1991: 79, Figs 85–86, Tab. 36); Balsamo et al. (2009: 08, appendix 1).

###### Material.

Soberbo: 2 specimens, Água limpa: 2 specimens, Preto River: 2 specimens, Gouveia: 2 specimens.

###### Description.

The description is based on a single adult specimen, 212.5 μm in total length. Head with oval edge and body long and wide. Body medium-sized, with head and neck weakly defined, but trunk and caudal base clearly distinct. Head with slightly five lobes and two pairs of ciliary tufts. Hypostomion weakly developed as a fine transverse furrow appearing as a thin line. Pharynx 56.25 μm in length from the posterior edge of the mouth to the pharyngo-intestinal junction, that lies at U26. Alternating columns of pedunculated, unkeeled, elongate scales along the body.

**Figures 1–9.**
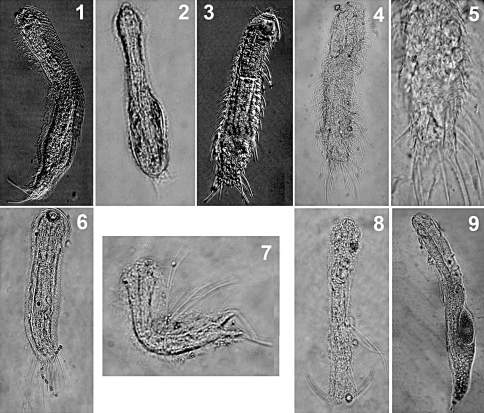
Freshwater psammic Gastrotricha from Brazil. **1** Aspidiophorus cf. pleustonicus: ventral view **2** Ichthydium cf. chaetiferum **3** Chaetonotus acanthocephalus: dorsal view **4** Chaetonotus heideri: dorsal view **5** Chaetonotus heideri: close-up of the dorsal scales **6** Chaetonotus sp. ventral view **7** Chaetonotus cf. succinctus: lateral view **8** Chaetonotus cf. succinctus: dorsal view **9** Redudasys sp.: dorso-lateral view.

**Table 1.** Morphometrical features of Aspidiophorus cf. pleustonicus. N= number of specimens measured.

Features	Range	N	Literature data from Brazil
Body length	212.5–275 µm	6	191–208 µm
Length of adhesive tube	31.25–37.5 µm	6	9.5–11.5 µm
Pharynx length	56.25–93.75 µm	6	22.6–27.2 µm
Diameter of mouth ring	11.25–18.75 µm	6	5 µm
Cephalion length	25.97 µm	6	-
Cephalion width	19.48 µm	1	16 µm

###### Remarks.

The genus Aspidiophorus counts 30 species in the world with 9 marine species and 21 freshwater (Balsamo et al. 2009) and in Brazil there are five freshwater species (Kisielewski 1991) and three marine (Todaro and Rocha 2004, 2005). Our specimens resemble Aspidiophorus pleustonicus Kisielewski, 1991 due to the long body, five-lobed head and shape of pedunculated scales. However, they can be distinguished from Aspidiophorus pleustonicus, from the size of the mouth, larger than in the original description (Table 1), and the absence of the cuticular rods internal to the pharynx.

###### Distribution.

Brazil: Diamantina, Gouveia (Minas Gerais State); São Paulo (São Paulo State).

#### Genus Ichthydium Ehrenberg, 1830

##### Ichthydium chaetiferum

(Müller, 1786) cf.

Fig. 2 Table 2

Ichthydium chaetiferum  – Kisielewski (1991: 81, Figs 87–89, Tab. 37); Hummon (2007: 10); Balsamo et al. (2009: 14, Appendix 1); Kånneby et al. (2009: 35).

###### Material.

Água limpa: 1 specimen.

###### Description.

The description is based on a single adult specimen, 108.49 μm in total length. Head with five lobes and two pair of ciliary tufts, with a pair of large “ocellar” granules. Pharynx 28.30 μm in length from the posterior edge of the mouth to the pharyngo-intestinal junction, that is at U26. 8 Spines present on the ventrolateral body side.

**Table 2.** Morphometrical features of Ichthydium cf. chaetiferum. N= number of specimens measured.

Features	Measures	N	Literature data from Brazil
Body length	108.49 µm	1	107–117 µm
Length of adhesive tube	16.98 µm	1	12.5–14 µm
Pharynx length	28.30 µm	1	25–29 µm
Diameter of mouth ring	4.01 µm	1	3 µm
Cephalion length	4.8 µm	1	8.5–9 µm
Cephalion width	9.6 µm	1	11 µm

###### Remarks.

The specimens collected in the present study resemble those described in Kisielewski (1991) due to the presence of a bulbous pharynx and “ocellar” granules. However, they can be distinguished from those in Kisielewski (1991) for the absence of the dorsal cephalic sensorial bristle and the number of spines in the ventrolateral body side (8 against 12).

###### Distribution.

Brazil: Diamantina (Minas Gerais State), Juréia Ecological Reserve (São Paulo State).

#### Genus Chaetonotus Ehrenberg, 1830

##### Subgenus Primochaetus Kisielewski, 1997

###### Chaetonotus (Primochaetus) acanthocephalus

Valkanov, 1937

Fig. 3 Table 3

Chaetonotus acanthocephalus  – Kisielewski (1981; 1991:54, Figs 65–68, Tab. 22); Hummon (2007: 5); Balsamo et al. (2009: 12, Appendix 1).

####### Material.

Água Limpa: 2 specimens, Soberbo: 1 specimens, Preto River: 5 specimens.

####### Description.

The description is based on an adult specimen, 236 μm in total length. Head with three lobes and a one pair of ciliary tufts. Five peculiar cephalic scales with long spines present on the head. Two ventral plates at the sides of the hypostomion. Pharynx 65 μm in length from the posterior edge of the mouth to the pharyngo-intestinal junction that is at U27. The general long-spine distribution pattern shows two pairs of conspicuous lateral neck spines. Two pairs of long lateral spines at the furcal base.

**Table 3.** Morphometrical features of Chaetonotus acanthocephalus. N= number of specimens measured.

Features	Range	N	Literature data from Brazil	Literature data from Europe
Body length	169–236 µm	2	123–175 µm	100–148 µm
Length of adhesive tube	27.5–28 µm	2	11–17 µm	14–16 µm
Pharynx length	62.5–65 µm	2	34–54 µm	37–49 µm
Diameter of mouth ring	8–8.75 µm	2	5 µm	6.5–8 µm
Cephalion length	20 µm	1	-	-
Length of neck scales	7 µm	1	6–7 µm	4–7 µm
Length of trunk scales	10 µm	1	5.5–9.5 µm	5.5–8 µm
Maximum length of the neck spines	24–27 µm	2	7.5–19.5 µm	11–15 µm
Maximum length of the trunk spines	30–35 µm	2	12.5–28 µm	16.5–22 µm
Length of terminal spines	19–31.25 µm	2	8–19.5 µm	-
Number of scales in a single longitudinal row	17	2	17	16–18

####### Remarks.

Kisielewski (1991) reported three distinct morphotypes of Chaetonotus acanthocephalus in Brazilian inland waters:two of them were collected in São Carlos city and one in Juréia Reserve. Our specimens appear to be close to one of the morphotypes found in São Carlos due to the presence of two pairs of long spines at the furcal base, and the peculiar transversal row of trunk spines (Kisielewski 1991:54, Figs 65–66). However, the body length, width and the posterior spines of the Diamantina specimens are larger than those observed from São Carlos (Table 3).

####### Distribution.

Brazil: Diamantina (Minas Gerais State); São Carlos (São Paulo State), Juréia Reserve (São Paulo State), Corumbá (Mato Grosso do Sul State); Poland: Lake Piaseczno; Germany; Bulgaria.

###### Chaetonotus (Primochaetus) heideri

Brehm, 1917

Figs 4–5 Table 4

Chaetonotus heideri  – Emberton (1981: 95, Figs 1–2); Balsamo (1990: 173); Kisielewski (1991: 17, Tab. 3); Nesteruk (2004: 444, Tab. 1; 2007: 836, Tab. 1); Grilli et al.(2008: 174, Fig. h).

####### Material.

Água Limpa: 1 specimen, Soberbo: 2 specimens, Preto River: 2 specimens.

####### Description.

The description is based on an adult specimen 137.5 μm in total length. Head with three lobes and two pairs of ciliary tufts. Pharynx 41 μm in length from the posterior edge of the mouth to pharyngo-intestinal junction, that is at U29. Anterior scales rounded and posterior ones pentagon-like shaped. Lateral spine denticle located near to the spine end.

**Table 4.** Morphometrical features of Chaetonotus heideri. N= number of specimens measured.

Features	Range	N	Literature data from Brazil	Literature data from Europe
Body length	137.5–137.96 µm	2	188–196 µm	106–220 µm
Length of adhesive tube	25–38.8 µm	2	22–25 µm	21–32 µm
Pharynx length	41 µm	2	48.5–50 µm	45–56 µm
Diameter of mouth ring	7.55–10.62 µm	2	10–11.5 µm	10.5–13 µm
Length of trunk spines	37.5–37.96 µm	2	22–37 µm	46–68 µm
Length of egg	11 µm	1	-	-

####### Distribution.

Brazil: Diamantina (Minas Gerais State), Juréia Ecological Reserve and São Carlos (São Paulo State), Benevides (Pará State); USA: Ohio; Germany; England; Italy; Poland; Romania; Russia; Czech Republic; Switzerland; France: Gironde.

##### Subgenus Lepidochaetus Kisielewski, 1991 [Balsamo et al. 2009, p.11]

###### Chaetonotus sp.

Fig. 6 Table 5 

####### Material.

Gouveia: 3 specimens.

####### Description.

The description is based on an adult specimen 236.95 μm in total length. Head with three lobes and one pair of ciliary tufts. Pharynx 64.93 μm in length from the posterior edge of the mouth to the pharyngo-intestinal junction (PhIJ), at U27. Hypostomion as a weak transverse furrow. Three pairs of lateral parafurcal spines, the two posteriormost longer than the adhesives tube. Adhesive tubes very long and thin.

**Table 5.** Morphometrical features of Chaetonotus sp. N= number of specimens measured.

Features	Range	N
Body length	150.76–236.95 µm	3
Length of adhesive tube	26.15–32.46 µm	3
Pharynx length	35.38–63 µm	3
Diameter of mouth ring	10–14 µm	3
Cephalion length	30.96 µm	3
Length of the egg	100 µm	3
Length of rearmost lateral spines	38.46–71.42 µm	3

####### Remarks.

The genus Lepidochaetus was originally described by Kisielewski (1991) to group some Chaetonotus species characterized by numerous, rounded, unkeeled scales, provided with long and thin spines covering both the dorsal and the ventral body surfaces. However, here we follow Balsamo et al. (2009) who considered this taxon as a subgenus of Chaetonotus. Our specimens resemble Chaetonotus (Lepidochaetus) brasilianus (Kisielewski 1991), due a similar scale shape and distribution, and rearmost lateral spines arranged in three pairs, which gradually grow in length in a caudal direction. However, they can be distinguished from the previously described species by the absence of cuticular rods and the neck sensorial bristles.

####### Distribution.

Brazil: Diamantina, Gouveia (Minas Gerais State).

##### Subgenus Zonochaeta Remane, 1927

###### Chaetonotus (Zonocheta) succinctus

Voigt, 1902 cf.

Figs 7–8 Table 6

Chaetonotus succinctus  - Anderson and Robbins (1980: 226); Kisielewski (1991, 60); Hummon (2007: 6); Weiss (2001: 313); Balsamo et al. (2009: 13, Appendix 1).

####### Material.

Cabral Mountains:1 specimen; Gouveia:1 specimen; Preto River: 1 specimen.

####### Description.

The description is based on an adult specimen 201.38 μm in total length. Head with five lobes and two pairs of ciliary tufts. Pharynx 55.48 μm in length from the posterior edge of the mouth to the pharyngo-intestinal junction, lying at U27. On the middle trunk region, a transverse band of five long dorsal spines, all terminally bifurcated, and of equal length (77.6 μm) and thickness. Paired spines at the furca base, not extending beyond the adhesive tube end.

**Table 6.** Morphometrical features of Chaetonotus cf. succinctus. N= number of specimens measured.

Features	Range	N
Body length	165.27–201.38 µm	2
Length of adhesive tube	41.6–43.05 µm	2
Pharynx length	54.1–55.55 µm	2
Diameter of mouth ring	4.13–6.89 µm	2
Length of trunk “band” spines	71.42–72.6 µm	2

####### Distribution.

Brazil: Diamantina, Cabral Mountains (Minas Gerais State), Belém (Pará State); Poland; Romania; England; Italy; Germany; South Korea.

Remarks. Within the subgenus Zonochaeta, four species (Chaetonotus bisacer, Chaetonotus cestacanthus, Chaetonotus dracunculus, Chaetonotus succinctus)are characterized by the presence of a series of long dorsal spines with concave apices (Balsamo 1990, 1999). In Brazil, only Chaetonotus succinctus and Chaetonotus bisacer were previously identified(Kisielewski 1991), and the main difference between the two species is the presence of a pair of long spines at the furca base, which extend beyond the adhesive tube tip.

### Order Macrodasyida Remane, 1925

#### Genus Redudasys Kisielewski, 1987

##### Redudasys sp.

Fig. 9 Table 7 

###### Material.

Água limpa: 8 specimens; Cabral Montains: 4 specimens. Video sequence (format .mov) is available at http://www.megaupload.com/?d=1F7NJ1XI

###### Description.

The description is based on an adult specimen 461.54 μm in total length. Cephalic cilia occur in one transverse dorsal row as well as in irregularly distributed tufts located at the anterolateral head margin. The mouth opening has a diameter of 10.1 µm. Pharynx 153.85 μm in length from the posterior edge of the mouth to the junction with the intestine. Two elongated caudal lobes, 25.64 µm long and 4.76 µm wide. Median caudal cone absent. Only anterior and caudal adhesive tubes are typically present. One anterior tube per side located laterally in the anterior part of the body. Seven tacticle bristles per side along the lateral body and one per side on the caudal end. Two pairs of caudal adhesive tubes. The inner tube (7.14 µm long) is usually 2/5 shorter than the external one (11.9 µm long).

**Table 7.** Morphometrical features of Redudasys sp. N= number of specimens measured.

Features	Range	N
Body length	280–461.54 µm	14
Pharynx length	87.5–153.85 µm	14
Diameter of mouth ring	10.1–11.0 µm	14
Length of external caudal tube	11.9–17.5 µm	14
Length of inner caudal tube	7.14–12.5 µm	14

###### Distribution.

Brazil: Diamantina, Cabral Mountains (Minas Gerais State).

###### Remarks.

The specimens found in Minas Gerais State areundoubtedly members of the genus Redudays, an incertae sedis Macrodasyida taxon recorded from a freshwater environment (Kisielewski 1987). However, the data gathered in the present study allow to exclude their affiliation to the single species described in this genus so far, Redudays fornerisae Kisielewski, 1987. Our specimens presented seven of tactile bristles per side along the body, and one per side on the caudal body end, that is a total of eight bristles per side, and in addition they had one anterior tube per side. Many of the great number of specimens found in our samples showed a large egg in the trunk region. The species Redudasys fornerisae presents six tactile bristles per side along the body and one per side on caudal body end, that is a total of seven bristles per side, and in addition they had two anterior tube per side. Furthermore, the description of Redudasys fornerisae was based on six adult individuals (Kisielewski 1987). Indeed, the specimen used for the description was considered an adult individual by the presence of mature oocytes and a mature egg. For this reason, we strongly believe that the specimen here described is an adult and cannot be considered as an early stage of development of Redudasys fornerisae. Moreover, even in the smaller specimens from the twelve specimens (0,280 mm; Table 7) there are eight tactile bristles per side.

## Discussion

The findings presented here allow us to draw some remarks on the Gastrotricha of the State of Minas Gerais. It is worthwhile noting that a poor sampling effort has allowed us to obtain very interesting faunistic data and to identify seven distinct species, which suggest a high biodiversity of Gastrotricha in this State. Up to now, 22 species of Gastrotricha Chaetonotida had been described from Brazilian rivers with slow water current and quiet habitats (Kisielewski 1991).

The most striking result of this study was the report of Redudasys specimens from different streams in Minas Gerais State. Thus, the discovery of Redudasys specimens outside of the original record is of great biogeographic interest, as the adaptation of this macrodasyidan taxon to the freshwater habitats could have been followed by considerable radiation, mainly in the neotropical region.

Albeit a high diversity of endemic gastrotrich chaetonotidans has been recorded in the Brazilian fauna (e.g. Undula, Arenotus - Kisielewski 1987, 1991; Balsamo et al. 2008), most of Gastrotricha species and genera found in Brazil have a cosmopolitan distribution. As pointed out by Kisielewski (1987, 1991) and Hummon (2007), species of the Chaetonotidae genera Aspidiophorus, Chaetonotus, Heterolepidoderma, Ichthydium, Lepidodermella, Polymerurus have a very wide distribution and are known from Europe, Asia, North and South America (Chaetonotus species were also collected in Africa and Oceania, and Polymerurus species in Oceania). Furthermore, up to now, 60 Chaetonotida species have been reported in Brazilian inland waters and 34 were also found elsewhere in the world, showing that 57% of the freshwater species have intercontinental or cosmopolitan distribution.

Based on preview studies (Kisielewski 1987, 1991; Todaro and Rocha 2004, 2005) and on our own results, it is important to emphasize that further investigations are needed to increase the knowledge of Brazilian gastrotrich fauna, which likely include a quite higher number of species.

## Supplementary Material

XML Treatment for Aspidiophorus pleustonicus

XML Treatment for Ichthydium chaetiferum

XML Treatment for Chaetonotus (Primochaetus) acanthocephalus

XML Treatment for Chaetonotus (Primochaetus) heideri

XML Treatment for Chaetonotus

XML Treatment for Chaetonotus (Zonocheta) succinctus

XML Treatment for Redudasys
